# Comment on Xu et al. Isolation and Identification of a Novel Phlebovirus, Hedi Virus, from Sandflies Collected in China. *Viruses* 2021, *13*, 772

**DOI:** 10.3390/v13122397

**Published:** 2021-11-30

**Authors:** Remi N. Charrel, Jerome Depaquit

**Affiliations:** 1 Unité des Virus Emergents, UVE, Aix Marseille University, IRD 190, INSERM 1207, 13005 Marseille, France; 2 ESCAPE EA7510, USC ANSES VECPAR, SFR Cap Santé, UFR de Pharmacie, Université de Reims Champagne Ardenne, 51096 Reims, France; jerome.depaquit@univ-reims.fr

## 1. Introduction

The article from Xu et al. entitled “*Isolation and Identification of a Novel Phlebovirus, Hedi Virus, from Sandflies Collected in China*” provides unprecedented results demonstrating that sandfly-borne phleboviruses are even more widespread than anticipated [1]. These results show that sandflies of China can be infected with novel phleboviruses as previously described for the Wuxiang virus, a close relative to the Sicilian virus [2,3,4,5]. However, some data presented in this study raise questions that are important to address in order to decipher the phlebovirus diversity in this geographic area of the world.

The technique used for determining the species of sandflies that are present in the pool is not convincing: monospecific pools of 50–100 sandflies have been constituted but it is not clear whether morphological identification was done by microscopic examination of head and genitalia after mounting each individual between the slide and cover slip or by observation under magnifying glass; the latter is likely to have been selected because it is more adapted to field conditions.

More data about the identification of the sandflies could have been proposed by Xu et al. It is not clear if females, males or both have been processed, and how many specimen(s) or batch(es) of sandfly(ies) have been found to be infected by Hedi virus. Regarding the identifications, five species have been recorded in the Shanxi province: *Ph. Chinensis*, *Ph. Mongolensis*, *Sergentomyia khawi*, *Se. squamirostris* and *Se. suni* [6]. Taking into account the lack of faunistic studies carried out in China, especially during the last 20 years, we consider the possibility that the distribution of sandfly species could be wider than initially determined. For example, in addition to the five species recorded in the Shanxi province, the following four additional species were recorded in the neighboring Shaanxi province: *Ph. stantoni*, *Ph. kiangsuensis*, *Se. nankingensis* and *Se. barraudi* [6]. The identification of females of the genera *Phlebotomus* and *Sergentomyia* is relatively easy for a trained entomologist, but their identification at the species level is impossible without individual mounting of the head and genitalia of each specimen between slide and cover slip and their examination under a microscope. On the other hand, there are four species of the subgenus *Adlerius* which have been recorded in China and, despite that *Ph. chinensis* is the only one officially recorded in the province of Shanxi, *Ph. chinensis* and *Ph. sichuanensis* could be found in parapatry [7] and the latter has been recorded recently in the vicinity of this province [8]. The taxonomy of the species belonging to the subgenus *Adlerius* is one of the most difficult within the phlebotomine sandflies. The identification of the males is based on a high-quality mounting between slide and cover slip of the genitalia exhibiting the parameral sheaths, as well as the aedeagal ducts which were sometimes very long (up to 1000 µm). Their identification without such mounting is impossible. Moreover, it is unanimously recognized that the identification of the females of this subgenus is absolutely impossible, despite the observation of well mounted specimens under a microscope at the highest magnification [9]. Consequently, it is necessary to focus on the sandfly identification by using appropriate molecular tools.

Morphological identification is highly time-consuming and might be difficult to adapt to field studies when downstream studies requiring sample preservation are planned. Recently, a new technique allowing retrospective species identification using a combination of cytochrome b and cytochrome c PCR subsequently analyzed through NGS has been described [10,11,12]. Briefly, comparing the reads with reference database sequences identifies whether the pool is mono- or poly-specific, and what are the respective proportions of each of the species constituting the pool. Although this does not provide undisputable evidence, this can clearly indicate whether viral RNA is derived from a unique species or possibly from different species. In the latter, analysis of >1 pools containing the same viral RNA statistical analysis of reads together with the combinatory analysis of read distribution in several pools can identify a unique species as the most likely vector. Mitochondrial PCR-NGS is useful to determine sandfly species content after pool is constituted; this technique can determine if (i) the pool is monospecific, (ii) or if the pool contains several species and (iii) identify the species within the pool. It is not helpful to constitute the pool; it can help to delineate the species content of one or several insect pools. Obviously, mitochondrial PCR-NGS will likely be performed on virus-positive pools only. However, it must be clear that if the pool consists of several species; this technique cannot identify which species of specimen contains the detected viral RNA. Unfortunately, Xu et al. have only used the cytochrome b PCR product that has been sequenced using the Sanger method and not NGS, which is the only technique that provide a sufficient number of reads to determine whether the pool consists of one or more species. For these reasons, the claim that the pool consists solely of *Phlebotomus chinensis* is poorly supported by the described method. 

In conclusion, we believe that the involvement of *Phlebotomus chinensis* as possible vector is very likely but still merits confirmation; in addition, the fact that other species belonging to the *Adlerius* subgenus might be involved in the transmission of Hedi virus should be considered.
